# Incomplete Uterine Artery Embolization Secondary to Collateral Parasitization from Celiac and Superior Mesenteric Arterial Branches: A Case Report and Literature Review

**DOI:** 10.3390/reports9040363

**Published:** 2026-10-20

**Authors:** Haidara Almansour, Mohammadhossein Ghasempourabadi, Darren Klass

**Affiliations:** 1 Division of Interventional Radiology, Department of Radiology, University of British Columbia, Vancouver General Hospital, Vancouver, BC V5Z 1M9, Canada; 2 Department of Neuroradiology, University Medical Center Mainz, Langenbeckstraße 1, 55131 Mainz, Germany; 3 Department of Radiology, University of Alberta, Edmonton, AB T6G 2B7, Canada

**Keywords:** uterine artery embolization, uterine fibroid, collateral circulation, superior mesenteric artery, celiac axis, parasitic arterial supply

## Abstract

**Background and Clinical Significance:** Uterine artery embolization (UAE) is a well-established treatment for symptomatic leiomyomas; however, treatment failure may occur owing to collateral arterial supply from extra-uterine sources. While ovarian artery collaterals are the most commonly reported cause, parasitic vascularization from the celiac axis and superior mesenteric artery (SMA) remains exceedingly rare and poorly documented; **Case Presentation:** A 41-year-old woman with symptomatic fibroids underwent technically successful bilateral UAE. Follow-up imaging revealed persistent vascularity of a dominant 19 cm pedunculated fibroid supplied by collateral branches from the celiac axis and SMA. As surgery was contraindicated owing to a recent pulmonary embolism that occurred after UAE, gonadotropin-releasing hormone (GnRH) agonist therapy was initiated, resulting in partial fibroid shrinkage and symptomatic improvement; **Conclusions:** Persistent fibroid vascularization after technically successful UAE may result from unrecognized extra-uterine arterial feeders. Cross-sectional angiographic imaging is essential for identifying atypical collateral pathways and guiding subsequent management in cases of treatment failure.

## 1. Introduction and Clinical Significance

Uterine artery embolization (UAE) is a well-established, minimally invasive, uterus-preserving treatment for symptomatic leiomyomas, with clinical success rates exceeding 80% and sustained long-term outcomes [1,2].

Collateral arterial supply, most commonly from the ovarian arteries, is a recognized contributor to UAE failure [3]. More rarely, parasitic vascularization may originate from branches of the inferior mesenteric artery (IMA) or the superior mesenteric artery (SMA), sustaining fibroid perfusion despite technically adequate embolization of the uterine arteries [3,4,5]. Chang et al. reported that IMA collaterals were present in 1.3% of UAE cases and were associated with larger uterine volumes and adenomyosis [4]. Dixon et al. described successful embolization of IMA collaterals following prior UAE failure [5], while Song et al. documented aberrant fibroid supply from SMA branches requiring selective embolization [3]. Cross-sectional vascular imaging, particularly contrast-enhanced MR angiography and CT angiography, plays a key role in detecting these atypical feeders and guiding re-intervention [6].

This case describes UAE failure due to parasitic arterial supply from both the SMA and celiac axis—a combination that, to our knowledge, has not been previously reported. We highlight the diagnostic and therapeutic challenges posed by extra-uterine collateral pathways and review the relevant literature.

Clinical Significance: This report documents a previously underreported pattern of incomplete embolization due to dual parasitic arterial supply to a uterine fibroid from the celiac axis and SMA following technically successful UAE. Recognition of such atypical collateral pathways is critical for avoiding treatment failure and guiding management in patients with large or pedunculated fibroids.

## 2. Case Presentation

**Figure 1:**
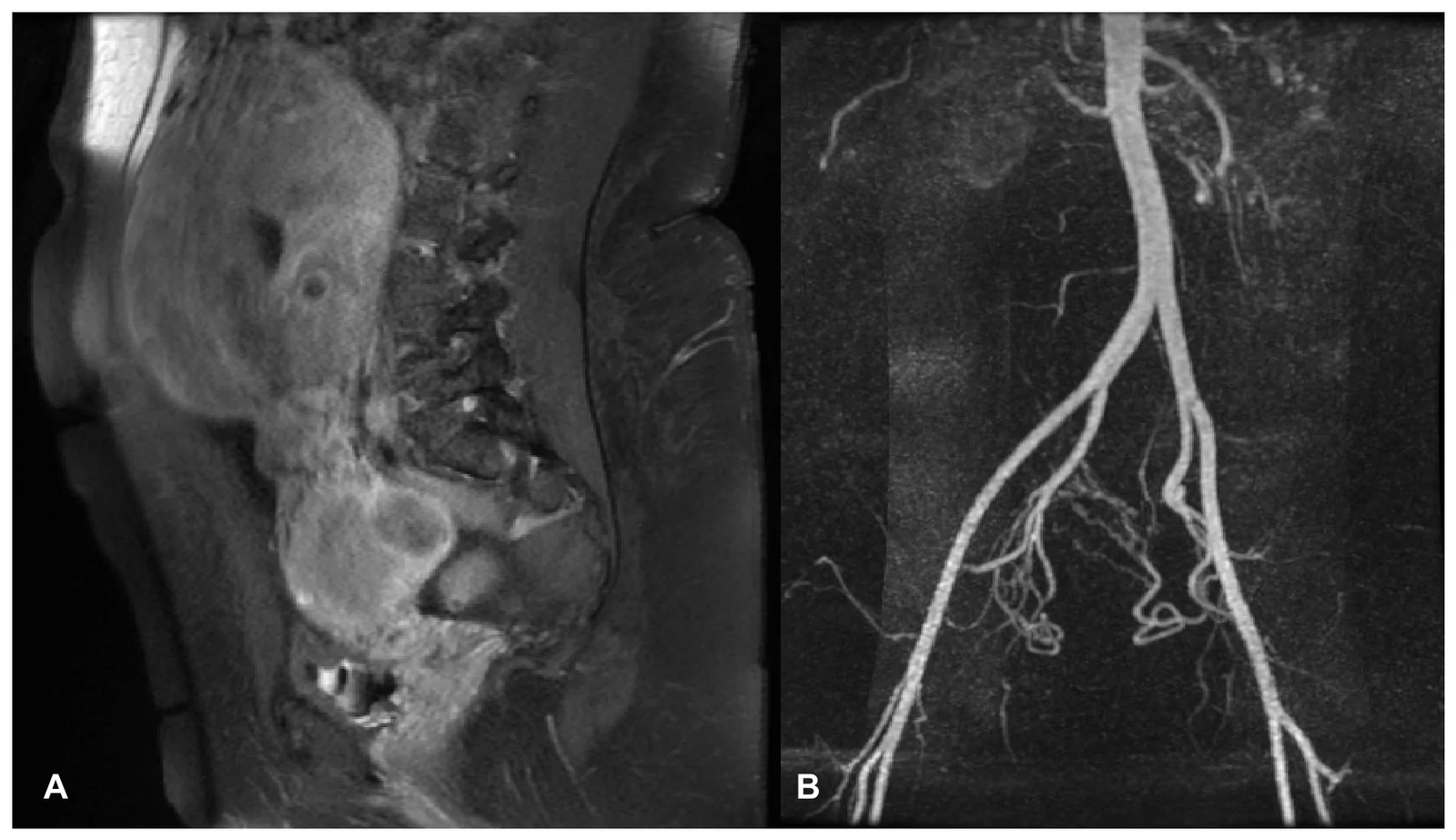
(**A**) Baseline T1-weighted post-gadolinium MR image in the sagittal plane showing a multifibroid uterus with a dominant 19 cm pedunculated fibroid arising from the uterine fundus. (**B**) Pre-procedural contrast-enhanced MR angiography demonstrating hypertrophied bilateral uterine arteries. No definite ovarian arterial supply is identified.

A 41-year-old premenopausal woman with a history of hypertension, morbid obesity and endometriosis presented with menorrhagia and pelvic pressure refractory to medical therapy. Magnetic resonance imaging (MRI) revealed a dominant 18.9 cm FIGO type 7 pedunculated fibroid arising from the uterine fundus, along with multiple smaller intramural fibroids (Figure 1). Pre-procedural contrast-enhanced MR angiography demonstrated hypertrophied bilateral uterine arteries with no detectable ovarian or other extra-uterine arterial supply to the fibroids (Figure 1). Given the patient’s preference, and that it has been shown that UAE is safe and effective even for giant fibroids [7], UAE was chosen over surgical treatment. Table 1 lists the advantages and disadvantages of both UAE and surgical treatment for a large pedunculated fibroid.

**Figure 2:**
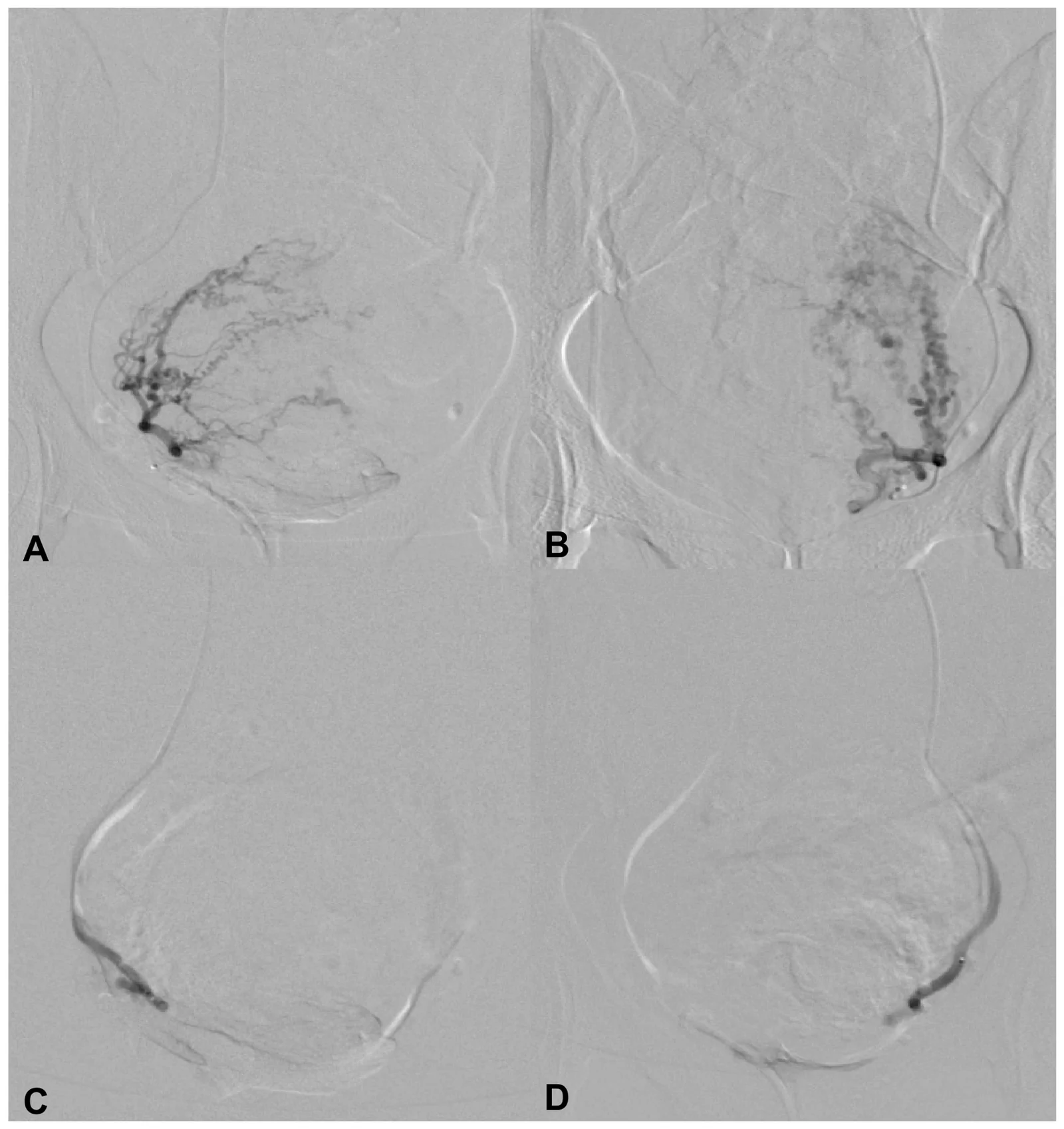
Intraprocedural digital subtraction angiography (DSA) images of the right and left uterine arteries before (**A**,**B**) and after (**C**,**D**) embolization. Pre-embolization images (**A**,**B**) demonstrate hypertrophied uterine arteries with prominent vascular supply to the fibroid. Post-embolization images (**C**,**D**) show technically successful embolization with markedly reduced arterial flow.

Via left distal radial access, each uterine artery was selectively catheterized with a 4-F catheter and a 2.9-F microcatheter. Intraprocedural digital subtraction angiography (DSA) confirmed hypertrophied uterine arteries with prominent vascular supply to the fibroid (Figure 2). Embolization was performed using 500–700 and 700–900 µm Embosphere microspheres. Post-embolization angiography demonstrated technically successful embolization with markedly reduced arterial flow and stasis in the uterine arteries, and no visible ovarian collaterals were identified (Figure 2).

**Figure 3:**
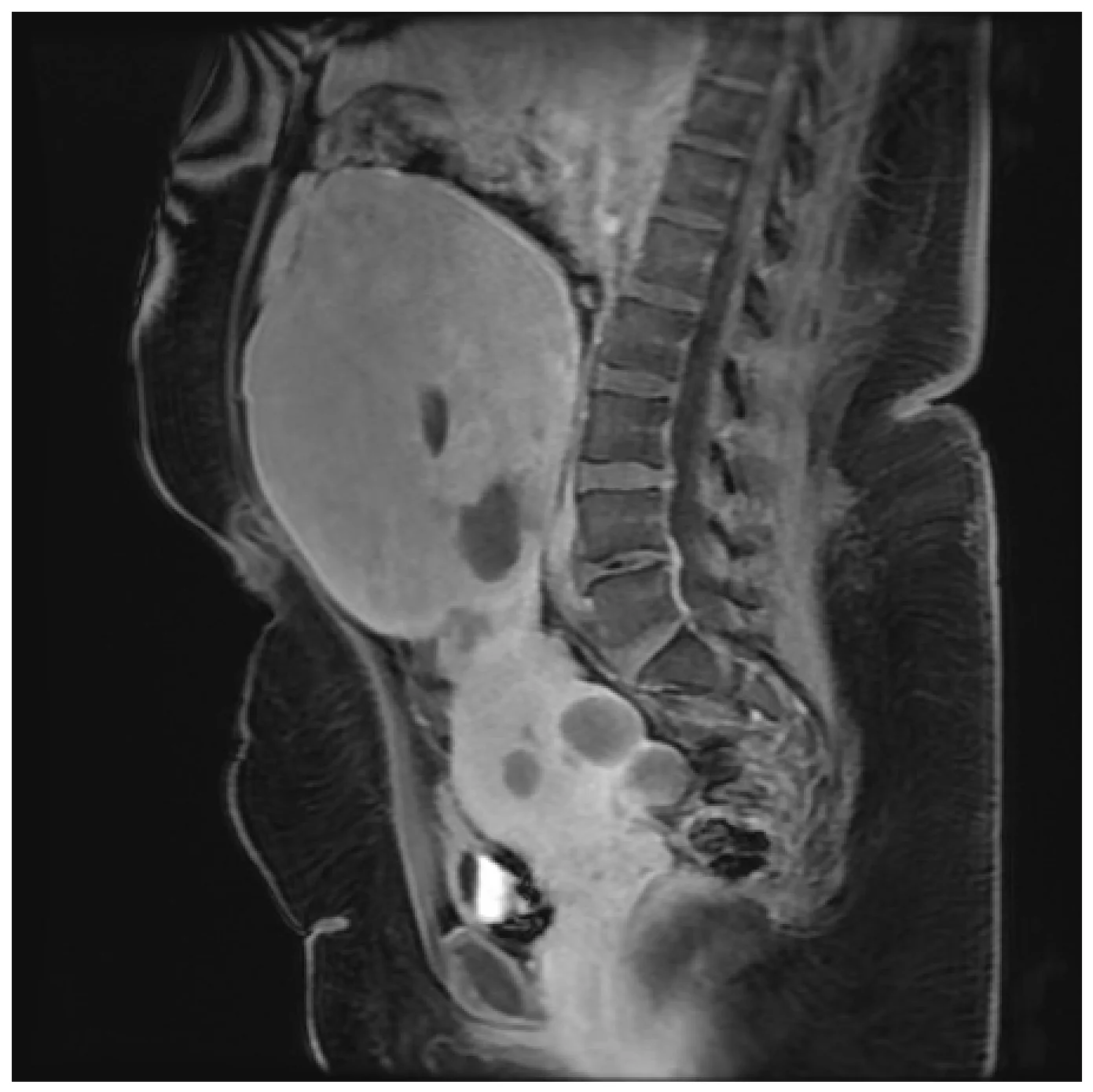
T1-weighted post-gadolinium MR image in the sagittal plane demonstrating successful devascularization of multiple intramural and submucosal uterine fibroids. In contrast, the dominant 19 cm pedunculated fibroid remains largely vascularized.

At the 3-month follow-up visit, the patient reported persistent menorrhagia and pelvic pressure. Post-embolization MRI demonstrated successful infarction of the smaller intramural and submucosal fibroids; however, the dominant 19 cm pedunculated fibroid remained largely vascularized with continued contrast enhancement (Figure 3). Thus, despite technically successful embolization of both uterine arteries, treatment of the dominant fibroid had failed.

**Figure 4:**
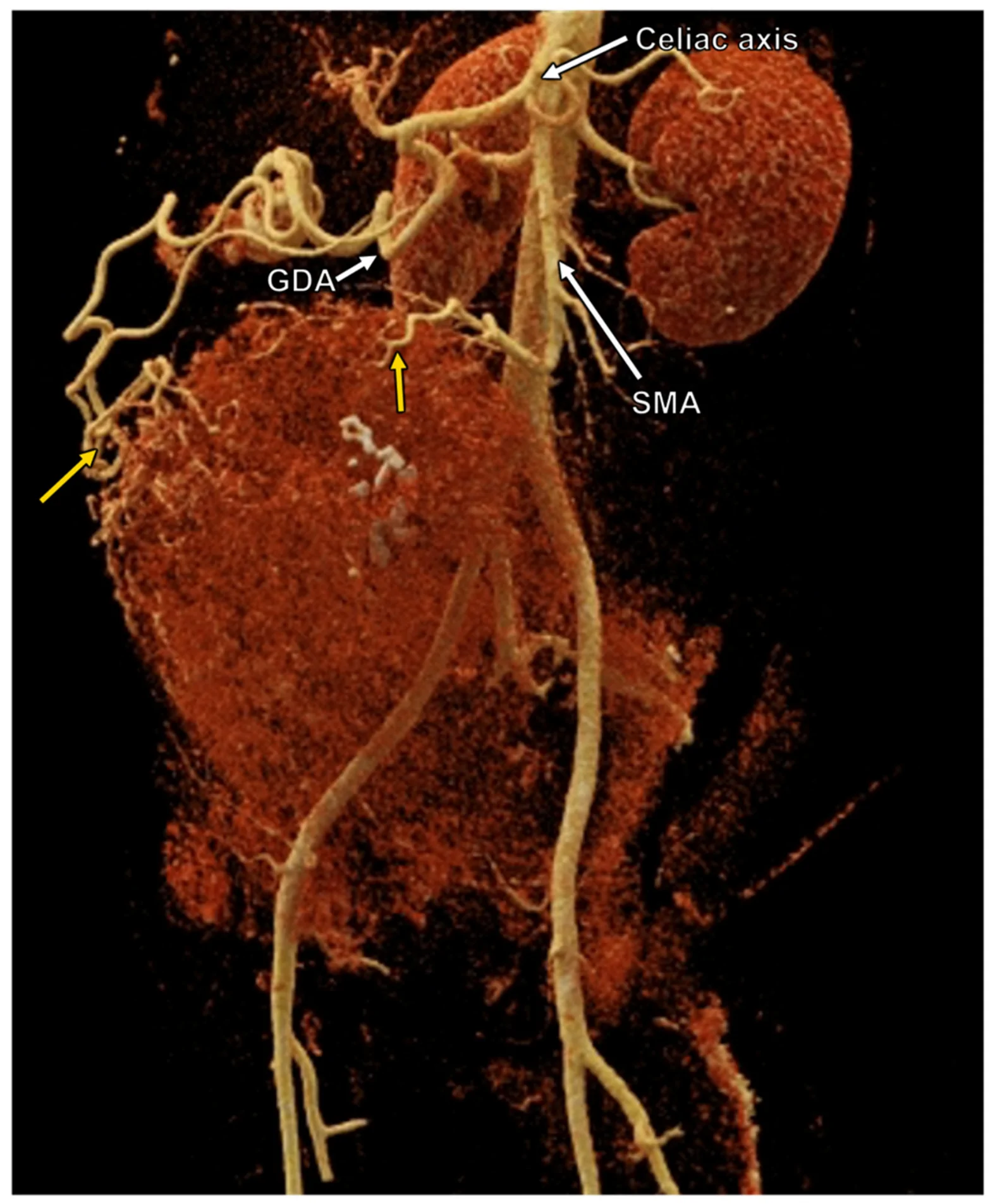
Three-dimensional volume-rendered CT angiography image, demonstrating aberrant parasitic arterial supply to the dominant pedunculated fibroid. Branches from the celiac axis and gastroduodenal artery (GDA) primarily supply the right and anterior portions of the fibroid, while branches from the superior mesenteric artery (SMA) supply its left portion. White arrows indicate the celiac axis, GDA and SMA; yellow arrows indicate the fibroid feeders.

Subsequent CT angiography revealed aberrant parasitic arterial supply to the dominant fibroid: branches from the celiac axis and gastroduodenal artery (GDA) primarily supplied the right and anterior portions, while branches from the SMA supplied the left aspect of the fibroid (Figure 4). These collateral pathways were not apparent on pre-procedural imaging.

Following UAE, the patient developed a pulmonary embolism unrelated to the procedure 4 months after UAE, for which therapeutic anticoagulation was started. Owing to elevated perioperative thromboembolic and bleeding risk following a recent pulmonary embolism, surgical intervention was deferred. Additional selective embolization was considered for preoperative devascularization before surgical resection, but was not pursued because of delayed surgery. Embolization alone was not deemed a sufficient treatment. The patient was started on gonadotropin-releasing hormone (GnRH) agonist therapy as a bridge to delayed surgery.

**Figure 5:**
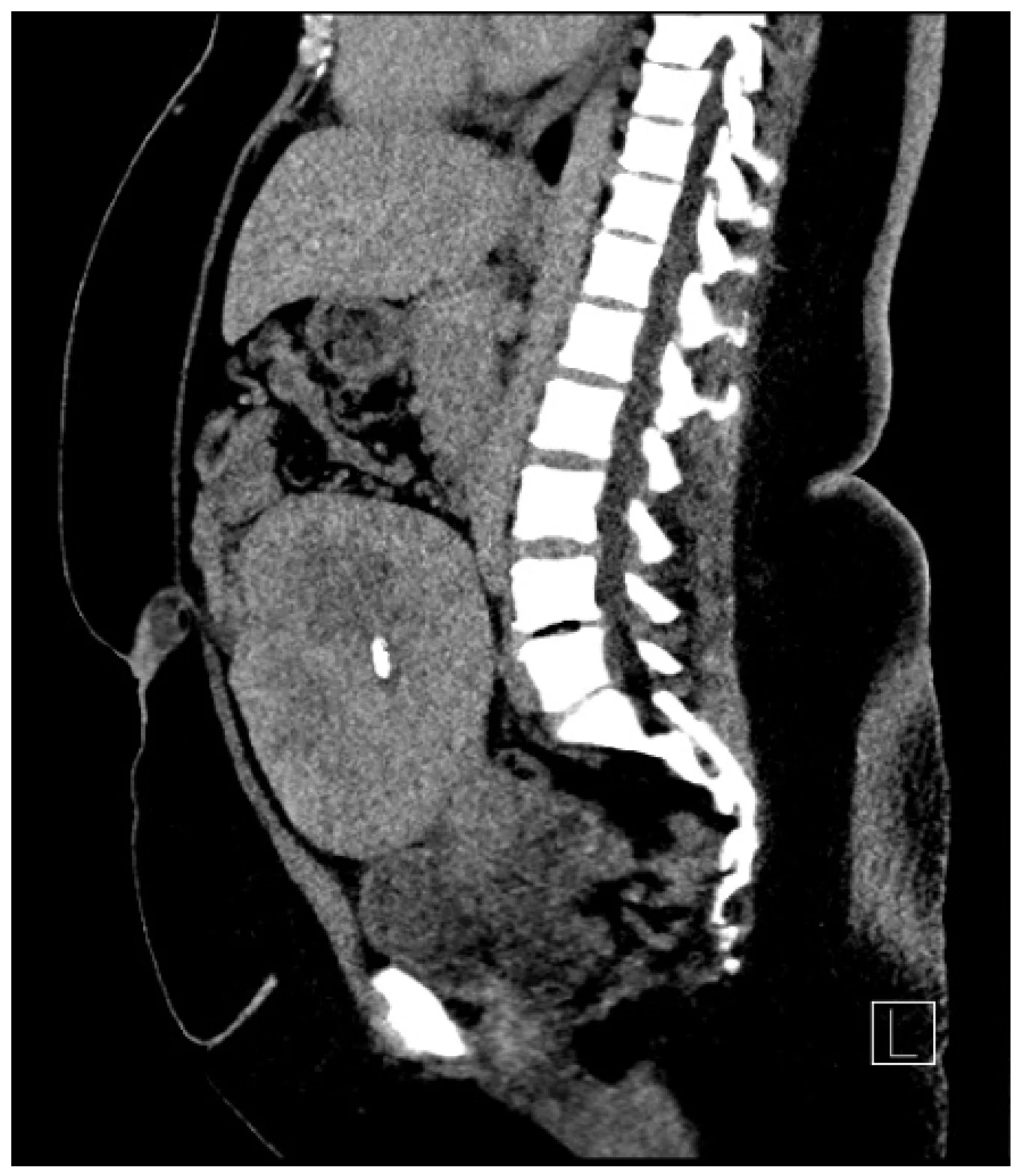
Follow-up contrast-enhanced CT sagittal image demonstrating interval reduction in the size of the pedunculated uterine fibroid to 15 cm following GnRH agonist therapy.

Follow-up contrast-enhanced CT demonstrated interval reduction in fibroid size to 15 cm, although persistent parasitic vascularity remained evident (Figure 5). The patient experienced partial symptomatic improvement, and surgical options continue to be reassessed.

## 3. Discussion

Uterine artery embolization is widely accepted as a safe and effective alternative to surgery for symptomatic fibroids, with clinical success rates approaching 90% in appropriately selected patients [1,2]. However, failure of symptom resolution following technically successful embolization raises concern for incomplete fibroid infarction, most often due to collateral blood supply. While the ovarian arteries are the most frequently identified source of extra-uterine perfusion, less common contributors include branches of the IMA and SMA [3,4,5].

This case illustrates a rare instance of UAE failure due to parasitic vascularization from both the celiac axis and SMA—a combination that, to our knowledge, has not been adequately described in the literature. Prior reports have documented aberrant fibroid supply from the IMA [4,5] and SMA [3] individually, and these collateral pathways appear to be recruited preferentially by large or pedunculated fibroids whose peripheral regions extend beyond the uterine arterial territory. In such cases, the uterine arteries may not represent the dominant vascular supply, and embolization confined to them alone may result in persistent fibroid viability.

Cross-sectional vascular imaging, particularly contrast-enhanced CT angiography and MR angiography, is crucial in evaluating treatment failure following UAE [6]. In this patient, CT angiography revealed persistent perfusion from gastroduodenal arterial branches—findings not apparent during the initial embolization procedure. Identification of such atypical feeders is essential for guiding further intervention, whether through targeted embolization of the collateral vessels or surgical planning.

Table 2 summarizes the published case reports and relevant studies on aberrant arterial supply contributing to UAE failure. As shown, collateral supply from the IMA is the most frequently reported non-ovarian source, while SMA feeders remain exceedingly rare. The current case adds to this limited body of evidence by documenting a dual collateral pattern involving both the celiac axis and the SMA.

This case underscores the need for heightened vigilance in post-UAE follow-up, particularly in patients with persistent symptoms and large pedunculated fibroids. Pre-procedural cross-sectional imaging may not always identify atypical feeders, as these collateral pathways can develop or become hemodynamically significant after embolization of the dominant uterine arterial supply. When technically feasible, selective embolization of identified collateral vessels should be considered; however, the anatomical complexity of celiac and mesenteric branches may limit this approach and necessitate alternative strategies such as hormonal therapy or surgical intervention.

Pedunculated subserosal fibroids were historically regarded as a relative contraindication to UAE; although later series showed acceptable safety and efficacy [7,10], large fibroids are more prone to extra-uterine collateral recruitment and incomplete infarction [4]. Surgery, such as laparoscopic occlusion of the uterine vessels with mini-laparotomy myomectomy, is therefore a valid alternative, and in a randomized trial myomectomy achieved better quality of life than UAE at 2 years [11]. The advantages and disadvantages of both approaches are summarized in Table 1. Regarding medical therapy, long-acting gonadotropin-releasing hormone (GnRH) agonists (e.g., leuprolide acetate, as used in this case) are considered effective but their hypoestrogenic effects limit their application and might necessitate hormonal treatment [12]. Oral GnRH antagonists (e.g., relugolix combination therapy) are also now available [12].

## 4. Conclusions

This case highlights a rare and previously underreported pattern of incomplete uterine fibroid embolization due to dual parasitic arterial supply from the celiac axis and superior mesenteric artery. It underscores the importance of considering atypical collateral pathways in patients with persistent symptoms following technically successful embolization, particularly those with large pedunculated fibroids. Cross-sectional angiographic imaging plays a critical role in identifying extra-uterine feeders and guiding further management. A multidisciplinary approach remains essential to tailor treatment strategies in complex cases involving aberrant vascular anatomy and elevated surgical risk.

## Figures and Tables

**Table 1:** Advantages and disadvantages of UAE and surgical treatment for a large pedunculated fibroid.

	UAE	Laparoscopic Vessel Occlusion with Mini-Laparotomy Myomectomy
Advantages	Minimally invasive; uterus-preserving; no general anesthesia; short recovery; might treat all fibroids in one session	Definitive removal of the dominant fibroid; histological diagnosis
Disadvantages	Lower efficacy in large or pedunculated fibroids; incomplete infarction from extra-uterine collaterals that may not be visible on pre-procedural imaging; possible re-intervention or adjunctive hormonal therapy	General anesthesia; blood loss from a hypervascular fibroid; longer recovery; perioperative thromboembolic risk; smaller intramural fibroids may remain untreated

**Table 2:** Case reports and relevant studies on aberrant arterial supply in UAE failures.

Year	Authors	Study Objective	Type of Collateral	Imaging Modality	Clinical Relevance	Key Findings
2014	Song et al. [3]	Case report: SMA parasitic supply to fibroids	SMA	CT angiography and conventional angiography	Illustrates rare SMA parasitic supply; underscores need for comprehensive vascular assessment after UFE failure	SMA branches can sustain fibroid perfusion; selective embolization required
2013	Salazar et al. [8]	Impact of embolizing utero-ovarian anastomoses on symptoms and amenorrhea	Utero-ovarian anastomoses	Angiographic identification of UOAs	Supports embolization of UOAs to reduce symptom recurrence; reassures regarding amenorrhea risk	OAE reduces recurrence when UOAs are present; no increased amenorrhea risk
2013	Chang et al. [4]	Prevalence and risk factors of IMA collaterals in UFE	IMA	MR and conventional angiography	Highlights IMA collaterals in large uteri or adenomyosis	IMA collaterals present in 1.3% of cases; associated with larger uterine volume and adenomyosis
2012	Dixon et al. [5]	Case report: IMA collaterals following UFE	IMA	Conventional angiography	Emphasizes need for follow-up imaging; re-intervention is common after UFE	Successful embolization of IMA collaterals after prior UAE failure
2010	Horton et al. [9]	Case report: anatomical variant of ovarian artery supplying the uterus	Ovarian (variant anatomy)	Conventional Angiography	Alerts to rare ovarian artery variants; reinforces need for detailed angiographic review	Unusual ovarian artery anatomy supplying both the uterus and adrenal gland

## Data Availability

All data are included in this published article. A preprint of this article was published on https://doi.org/10.21203/rs.3.rs-7172065/v1.
